# 761 Transforming Fluid Resuscitation Documentation to Improve Quality Outcomes

**DOI:** 10.1093/jbcr/irae036.303

**Published:** 2024-04-17

**Authors:** Shawna A Thomas, David Roggy, Natalie Fitzgerald, Kim Tosino, Dennis Magbanua, Cortni Grooms, Allison N Boyd

**Affiliations:** Eskenazi Health, Indianapolis, IN; Richard M. Fairbanks Burn Center at Eskenazi Health, Indianapolis, IN; Eskenazi Hospital, Indianapolis, IN; Eskenazi Health, Zionsville, IN; Eskenazi Health, Indianapolis, IN; Richard M. Fairbanks Burn Center at Eskenazi Health, Indianapolis, IN; Eskenazi Hospital, Indianapolis, IN; Eskenazi Health, Zionsville, IN; Eskenazi Health, Indianapolis, IN; Richard M. Fairbanks Burn Center at Eskenazi Health, Indianapolis, IN; Eskenazi Hospital, Indianapolis, IN; Eskenazi Health, Zionsville, IN; Eskenazi Health, Indianapolis, IN; Richard M. Fairbanks Burn Center at Eskenazi Health, Indianapolis, IN; Eskenazi Hospital, Indianapolis, IN; Eskenazi Health, Zionsville, IN; Eskenazi Health, Indianapolis, IN; Richard M. Fairbanks Burn Center at Eskenazi Health, Indianapolis, IN; Eskenazi Hospital, Indianapolis, IN; Eskenazi Health, Zionsville, IN; Eskenazi Health, Indianapolis, IN; Richard M. Fairbanks Burn Center at Eskenazi Health, Indianapolis, IN; Eskenazi Hospital, Indianapolis, IN; Eskenazi Health, Zionsville, IN; Eskenazi Health, Indianapolis, IN; Richard M. Fairbanks Burn Center at Eskenazi Health, Indianapolis, IN; Eskenazi Hospital, Indianapolis, IN; Eskenazi Health, Zionsville, IN

## Abstract

**Introduction:**

Accurate and readily available documentation of burn fluid resuscitation is essential for decision-making by the multidisciplinary team during the first 24 to 48 hours of care. Fluid resuscitation documentation in the electronic medical record (EMR) is ideal. However, due to the limitations of the EMR, bedside nurses must navigate to various tabs to document fluids, view orders, document events, review labs, and notify providers. To decrease burden of care and improve the accessibility of accurate fluid resuscitation information, our multidisciplinary team embarked on a journey to create a new format in the EMR for fluid resuscitation with our information technology (IT) department.

**Methods:**

In April 2022, the burn team initiated discussions to build a new and streamlined process for documentation of fluid resuscitation. The goal was to decrease the amount of navigation in the EMR during fluid resuscitation by the bedside nurse and improve the accessibility of meaningful hourly documentation for the multidisciplinary team.

During monthly meetings, a multidisciplinary team of nurses, a pharmacist, educators, and IT professionals collaborated on each component needed to capture the desired detailed documentation of each fluid resuscitation.

**Results:**

The new format for fluid resuscitation documentation in the EMR is constructed as a navigator flowsheet and initiated in September 2023. The bedside nurse performs all documentation on this one flowsheet. Information from the Lund-Browder calculates initial fluid resuscitation rates and goal hourly urine output. Once the bedside nurse initiates the fluid resuscitation, navigating to any additional flowsheets or tabs in the EMR is unnecessary. Certain information also pulls in automatically from devices, such as vitals, bladder pressure, and urine output.

Documentation of hourly fluids infused, vital signs, urinary output, fluids changes, bedside procedures performed, provider notification, nursing notes, and consulting team interventions are all performed in the narrator. The bedside nurse can also access all orders, labs, and notes without navigating elsewhere in the EMR. Real-time documentation using the narrator allows multidisciplinary team access without disrupting the patient’s nursing care. The post-resuscitation report provides hour-by-hour documentation for debriefing and reviewing each fluid resuscitation.

**Conclusions:**

The development of this new tool has afforded bedside nurses more time spent caring for the patient rather than navigating the EMR. Providers no longer go to the patient room to retrieve information to help guide fluid resuscitation and disrupt patient care. The post-resuscitation report provides an accurate account of the fluid resuscitation period for debriefing and review.

**Applicability of Research to Practice:**

Our process can be shared with other burn centers hoping to replicate this process.

